# Parents’ Educational Background and Child’s Learned Skills Are More Predictive for a Positive School Career than Earlier Parenting Behavior or Child’s Mental Health—Results from an 18-Year Longitudinal Observation Study

**DOI:** 10.3390/children12040506

**Published:** 2025-04-15

**Authors:** Beate Muschalla, Ann-Katrin Job, Wolfgang Schulz

**Affiliations:** 1 Psychotherapy and Diagnostics, Technische Universität Braunschweig, 38106 Braunschweig, Germany; wolfgang.schulz@tu-braunschweig.de; 2 Institute of Psychology, University of Kassel, 34127 Kassel, Germany; a-k.job@uni-kassel.de

**Keywords:** educational and professional degree, young adults, capacities, mental health, prediction, longitudinal study

## Abstract

**Background/Objectives**: Developmental research has shown that mental health and functioning is determined by social background and child and family characteristics. Until now, there have been few longitudinal studies which considered several aspects at the same time and observed children’s development over ten or more years. **Methods**: The aim of this 18-year-longitudinal study is to find out to which degree different child, family, and socioeconomic factors during early childhood (4 years of age) are associated with educational and professional outcomes in young adulthood (22 years of age). Of the initial sample of 280 participating families, 225 could again be investigated with standardized interviews and questionnaires at the 18 years follow-up (retention rate: 80%). **Results**: Educational degree of the parents was predictive of the child’s school success (*β* = −0.267, *p* < 0.001, in regression analysis). Maternal mental health (*β* = −0.005, *p* = 0.953), parenting behavior (*β* = −021, *p* = 0.782), and early child mental health problems (*β* = 0.071, *p* = 0.551) only had a low impact. The child’s sex did not predict school success. Better early learned skills (i.e., crystalline intelligence), but not cognitive skills, as measured by the child-specific intelligence test K-ABC, made children more likely to achieve good school-leaving grades (*β* = −0.240, *p* = 0.008). Children’s early mental health problems had no relevant impact on school degree (*d* = 0.00, *p* = 0.934/*d* = 0.02, *p* = 0.52 3) or professional status (*d* = 0.04, *p* = 0.157/*d* = −0.02, *p* = 0.299) at age 22. **Conclusions**: Besides the not-changeable parental education level, (learnable) competency aspects may be more predictive of a child’s educational success until young adulthood than earlier mental health problems in parents and children. This is good news as it supports the idea that mental health deficits can be compensated for through learning and competency training.

## 1. Introduction

### 1.1. What Predicts School and Professional Performance?

Many social, emotional, and cognitive school-relevant capacities are developed in early childhood [1,2]. In addition, various individual child, family, and socioeconomic factors during early childhood are associated with later academic performance, school completion, and career prospects [3,4,5]. The strongest influence on an individual’s school performance is exerted by cognitive and metacognitive capacities and prior knowledge [5], as well as social–emotional competencies [6]. On the side of non-cognitive characteristics student’s motivational [7] and classroom characteristics [8] as well as family conditions [9] are considered predictive. Sociodemographic, school, and educational factors seem to have somewhat weaker effects [8]; however, in some countries, the socioeconomic family background is highly related to children’s school success [10,11], and of course the quality of school impacts on students competency development [12].

Research so far has often focused on specific predictors for healthy development [13] or specific achievements [14,15]. Thus, there is still a need for evidence of broader developmental prediction perspectives which include aspects of family characteristics as well as aspects of child and parental mental health and behavior, to find out which aspects are more or less important for broader school success in young adults.

Accordingly, the aim of this 18-year-longitudinal study was to examine differentiated aspects of family characteristics, mental health, and behavior in parents and their young (4 years old) children, to find out which of them are predictive of children’s school and professional success at 22 years of age.

### 1.2. State of Research on Predictors for School and Professional Performance

In the following, we summarize the empirical knowledge on the core predictors for school performance and professional performance in adolescence and young adulthood. These person and context factors have been assessed in this present longitudinal study, in order to examine their predictive value for school and professional performance outcomes in young adulthood.

#### 1.2.1. Socioeconomic Status (SES) of the Family

School performance studies such as PISA [11], TIMSS [16], and LOGIK [17] consistently show that SES and school performance are directly related. For over 80 years, international results have also pointed to the relationship between SES and school performance [18,19]. A key finding of the PISA studies is that educational success is strongly related to SES in some countries [11]. Although the influence of socioeconomic factors on school success has decreased from 2003 to 2012, it still plays a crucial role [11]. It can thus be expected that children from families with high SES have better school achievements [20,21,22,23,24,25]. In the present study, we assessed the monthly household income of the family and the educational level of the parents at the first assessment (Pre) as indicators for SES.

#### 1.2.2. Parenting Behavior and Parental Mental Health

The quality of the early family environment is particularly formative for intellectual and psychosocial development [3]. For children, the family not only represents a developmental and living space but also an important learning environment. In this context, the importance of parenting behaviors such as warmth, praise, responsiveness, and parental school engagement for positive school-related behavior in children has repeatedly been discussed [23,26]. Dysfunctional parenting behaviors during early childhood, in turn, have been shown to be associated with worse school performance in children at about 6 years of age [27,28].

Parental mental health problems also seem relevant in the sense that they—due to genetic reasons, or model learning, or parents’ problems in socio emotional childcare—can affect child’s mental health and result in problems that are relevant for children’s school achievement [29,30,31,32,33,34,35].

#### 1.2.3. Positive Parenting Programs (e.g., Triple P)

The Triple P is a well-established evidence-based preventive parenting program that trains parents how to positively interact as a family and with their very young children. The aim of such parent training is to strengthen the parental relationship and parenting skills to promote the healthy development of children. Several self-reflection exercises are used to train positive parental behavior [36,37].

According to these empirical findings, parental (maternal) mental health problems, dysfunctional parenting behavior, and participation in preventive parent training were assessed as potential predictors for child’s school and professional outcomes.

#### 1.2.4. Child’s Sex

The sex difference in academic performance is most salient in language learning. In almost all European countries, girls usually perform better than boys of the same age in language subjects [11,38,39]. In contrast, boys’ academic performance in mathematics is usually better than girls’ [11,38]. Corresponding results are provided by a five-year longitudinal study of over 70,530 English children [40].

In the present study, we consider the sex of the child as a control variable in the prediction of later school success. As girls might achieve better grades in language subjects, but boys might achieve better grades in mathematics, the overall school success should not be dependent on the sex of the child.

#### 1.2.5. Cognitive Capacities and Learned Skills

Cognitive functioning is a main aspect of health over the life span and is often determined by high education levels in youth [11]. Cognitive skills such as intelligence and attention are cited as relevant cross-domain competencies for school success [40,41]. A longitudinal study of 101 children demonstrated that cross-domain precursor skills such as intelligence and working memory performance in preschool age are related to children’s performance in early school years [2]. In intelligence research, fluid intelligence and crystalline intelligence are distinguished. Fluid intelligence (capacities), on the one hand, encompasses basic processes of thinking and is largely independent of experience; it is very strongly genetically determined. Crystalline intelligence (learned skills), on the other hand, comprises the ability to apply acquired knowledge; it is considered to be predominantly culture-dependent [42].

In the present study, we assessed fluid capacities and learned skills as two dimensions of cognitive capacities in early childhood, and both are investigated as potential predictors for school and professional success in young adulthood.

##### Behavioral Problems, Mental Health, and Self Control

It has often been observed that self-control and behavioral or emotional problems during early childhood are related to later achievement [30,43,44,45,46]. Children’s socio-emotional competencies and psychological behavioral problems are related to preschool competencies and later school performance [47,48,49,50,51]. For example, in one study, children with externalizing behavior problems during early childhood showed deficits in cognitive and preschool skills at age three [49]. The impact of psychopathology-related behavior problems on school performance was also investigated [47]: An initial survey of 1514 children assessed anxiety and emotional disturbance, depressive symptoms, and obsessive–compulsive disorders. The following year, 563 children were found to comprise an at-risk group and underwent a diagnostic interview to assess behavioral problems. Two years later, school performance could significantly be predicted by earlier mental health symptoms and behavioral problems. Especially, ADHD symptoms had a negative influence on school performance, as well as depressive symptoms and persistent anxiety. Social anxiety and generalized worrying, in turn, positively predicted school performance. The regression model explained a total of 27% of language achievement and 19% of math achievement.

In the present study, we dimensionally assessed child mental health problems by using observer reports from the mothers.

#### 1.2.6. Adverse Childhood Events (ACEs) and Bullying

Adverse childhood events (ACEs) and bullying in youth may have a negative impact on children’s school success and professional career. Until know, longitudinal research suggests that ACEs may influence children’s behavioral and academic outcomes early in development [51]. Bullying is a frequent problem and reaction to the behavioral problems of children with observable neurocognitive or mental health problems [52]. Empirical findings show that bullying often occurs repeatedly, at school and later at work [53]. Therefore, in the present study, bullying in youth and ACEs were investigated as potential predictors for later school and professional problems in young adulthood.

### 1.3. Excursus: The German School System

In Germany, school attendance is obligatory for children and young people from the age of 6 until the end of the 9th school year. Attending state school is generally free for children and young people. Private schools, e.g., those run by church organizations, are only attended by very few children, even though there has been an increase in the last 5 to 10 years. The German school system is divided into primary, lower secondary, and upper secondary school depending on age and learning progress. The primary level is attended by all children regardless of their performance and lasts four or six years. In contrast to the school systems in many other countries, where all children, regardless of their performance, attend the same type of school at lower secondary level, most children in Germany attend a three-form school system with different school-leaving certificates. Depending on their performance level in primary school, children attend either Hauptschule (up to grade 9), Realschule (up to grade 10), or Gymnasium (up to grade 10 or 13; the last 3 years are also known as upper secondary level). There is a certain degree of mobility between the individual school types and, therefore, the possibility to switch between them. In addition, there have been more Integrative Gesamtschulen (inclusive comprehensive schools) in recent years, which can be attended by all children regardless of their performance in primary school, where all types of school qualifications are possible. With very few exceptions, there is no correlation between school quality and parental income [54].

### 1.4. Research Question

This aim of the present 18-year-longitudinal prediction study is to find out to which degree individual child-, family-, and socioeconomic factors during early childhood (4 years of age) as well as adverse childhood experiences (ACEs, up to age 18) and bullying in adolescence (at 14 years) are associated with educational and professional achievement in young adulthood (22 years of age). Because part of the families participated in the universal preventive Triple P Positive Parenting Program at the beginning of this study (for details, see Hahlweg and Schulz [55]), we also investigate the impact of program participation on school and professional success.

Although there is a large number of cross-sectional studies on the importance of family background for children’s school and professional success, there is still a lack of longitudinal studies demonstrating the long-term effects over a long period of time. Particularly in Germany, with its three-form school system that differs from that of most other countries, there is a lack of longitudinal studies. The present study aims to close this research gap.

Based on the reported state of research, it will be explored to which degree school and professional success of young adults is determined by

- Educational level of parents and household income;
- Parenting behavior and approximate health;
- Parental participation in a preventive parenting program (Triple P [36]) 18 years ago;
- Cognitive capacities, self-control, and early mental health problems in the child;
- Problems with ACEs and bullying in the child.

## 2. Materials and Methods

A large longitudinal observation study was conducted with families from a German city over 18 years. This study repeatedly assessed child and family characteristics as well as child behavioral problems from early childhood at the age of 4 years (Pre) over adolescence (10-year follow-up, FU10) to young adulthood (18-year follow-up, FU18). Risk and protective factors were assessed in early childhood (Pre), bullying was assessed in youth, and mental health problems, ACEs, and school achievement were assessed in young adulthood (FU18).

### 2.1. Recruitment

Since most children at 4 years of age attend day care centers (“kindergarten”) in Germany, recruitment was conducted via day care centers in the city. In the year 2001/02, all 33 day care centers (kindergarten) in the city of Braunschweig were informed about the research project, and 23 expressed their interest in participating. Out of the interested, 17 (i.e., 51.5% out of all the 33 day care centers) were randomly selected for family recruitment. The final eligible population from these 17 participating day care institutions included 915 families, of whom 280 families agreed to participate. The recruitment rate of 30.6% can be rated as very good for a universal prevention study. However, during the successive recruitment process, we observed that in areas of the city with lower socioeconomic level, the recruitment rate was lower than in socioeconomically higher-level areas. The non-participating parents were younger and more likely to receive state financial support. Parents who declined to participate usually feared an intrusion into their private lives, e.g., they expected the interview to be too personal, they did not want to be analyzed, or video recorded, or simply stated that they did not have time to participate in this study. Detailed information on the recruitment process and reasons for non-participation are reported by Bertram et al. [56].

Since participation of the families was conducted free-willingly, we expect that the participating families were interested in the research topic and somehow open for new experience. Families with a higher education background were over-represented (about 60% of parents had A-Levels) as compared to what would normally be expected in the general population. Due to these reasons, the sample represents a rather prototypically representative population, i.e., families with potentially better resources (socioeconomic background and education level). This limitation will be considered when interpreting the data.

At the Pre stage, each family received EUR 50 for their study participation. During the follow ups, the interviewed parent and the young adult each received EUR 40 (FU10) or EUR 50 (FU18) for participating in the approximately 2.5 h survey.

### 2.2. Instruments

Data were collected by using a combination of interviews and standardized questionnaires for children and parents (Table 1). The interviewers were prepared for the interviews with the families in advance through intensive training (12 to 15 h for each interviewer) by the project management. The training included role playing according to case vignettes, dealing with answers which are difficult to code, and assuring a private atmosphere, in order to keep the interviewed person motivated to answer all questions. The training’s aim was to make sure all interviewers adhere to the interview manual, in order to collect reliable data. The questionnaires were completed with paper and pencil or at FU18 online via SurveyMonkey (www.surveymonkey.de, 4 April 2025).

At baseline (Pre), when children were on average 4 years old, socio-demographics of the family were assessed by personal interviews (for details see Table 1). Child’s characteristics in terms of self-control problems, mental health, and intelligence, as well as parenting behavior and mothers’ self-reported mental health problems, were assessed by standardized tests and questionnaires (Table 1).

At FU10, when the children were on average 14 years old, family socio-demographics, the children’s school achievements, and whether the child was involved in bullying was assessed (Table 1).

At FU18, when the children were on average 22 years old, again, family socio-demographics were assessed, as well as the children’s ACEs, school-leaving achievements, and professional status (Table 1).

### 2.3. Statistical Analysis

First, for a basic overview of the descriptive data, means and percentages on all sample characteristics are reported (Table 2). Second, for a basic view on associations between predictors (family’s characteristics) and outcomes (school-leaving degree, professional degree, and overall school grade), correlations were calculated (Table 3).

Third, hierarchical regression analyses (Table 4, Table 5 and Table 6) were conducted, to estimate the relative predictive value of each predictor by including all relevant predictors according to the research question. Depending on the level of the outcome variable, either multiple linear regression (for continuous outcome variable, Table 6), or multinomial logistic regression (for categorial outcome variable, Table 4 and Table 5) were calculated. Five models were calculated for each of the hierarchical regressions, with additional variables added at each level (see Table 4, Table 5 and Table 6).

Our hierarchical structure is based on the following logic:

Level 1: socio-demographic data (Pre);
Level 2: psychological characteristics of the child (Pre);
Level 3: characteristics of the mothers (Pre);
Level 4: Triple P intervention (Pre);
Level 5: psychological characteristics of the child (FU10 and across the lifespan).

The multiple coefficients of determination R^2^ were interpreted according to Cohen [63]: R^2^ > 0.02 is interpreted as low variance clarification, R^2^ > 0.13 is interpreted as moderate variance clarification, and R^2^ > 0.26 is interpreted as high variance clarification. Interpretation of the effect sizes (Cohen’s d) and ß values is as follows: A Cohen’s d/ß value of 0.20–0.50/0.10–0.30 is interpreted as a small effect, a value of 0.50–0.80/0.30–0.50 is interpreted as a medium effect, and a value of >0.80/>0.50 is interpreted as a large effect; r > 0.10 is interpreted as a small effect, r > 0.30 is interpreted as a medium effect, and r > 0.50 is interpreted as a large effect 0.57.

Almost all multinomial logistic regressions showed sufficient model quality (LQ-Test p significant; Goodness of Fit (except the first model for professional degree which was not significant)). For the dependent variable school degree >82.5% of the young adults were correctly assigned by the model, and for professional degree, the rate was >62.8%. Nagelkerkes R^2^ was acceptable or good for all models concerning school degree (>0.297), and acceptable for professional degree (>0.245; except the first model therein). To verify multicollinearity, on the one hand, correlations between the twelve predictors were calculated and, on the other hand, the VIF values were determined [64]. None of the 66 correlations are >0.80; the VIF values fluctuate between 1.01 and 3.09 (<10). It can, therefore, be assumed that there is no multicollinearity between the predictors. Variance clarification (R^2^) was moderate; all models were significant.

## 3. Results

### 3.1. Sample Characteristics

The initial sample consisted of 280 families. Based on a randomized assignment, nearly half (48.6%) of the families received a brief parenting intervention (Triple P, [36]) after baseline assessment as part of the experimental condition (for details, see Hahlweg and Schulz [55]). Parents allocated to the control group were not provided with any training. In both groups, changes were observed over the time of data collection. At the first assessment point (Pre), the children were on average 4.1 years old (SD = 1.0). For the 10-year follow-up (FU10), 249 families could still be recruited (retention rate: 89%). At this time, the adolescents were on average 14.4 years old (SD = 1.2). In the 18-year follow-up (FU18), 225 families still participated (retention rate: 80%). The reasons for dropout were as follows: only about 5% of families refused to participate after being contacted by email or telephone, 10% could not be contacted in person, although contact details seemed to be up to date, and 5% could not be located at all.

The following analysis has been conducted with the FU18 sample.

The FU18 sample of young adults consists of 49.3% women and 50.7% men. The mean age was 22.6 years (SD = 1.1). The educational level of the parents was slightly higher than in the general population: in our sample, about 60% of parents (Pre) had the highest possible school degree (A-Levels/high school, 12 years completed; Table 1), but there were also about 10% without a school-leaving certificate. At FU18, 79.9% of the young adults had a school-leaving degree with 12 or 13 completed school years (high school level). Moreover, 59.9% had already finished a college degree or were in a study program at college. Thus, in our sample, there is enough variance of persons with the highest and lowest education, but the distribution is slightly different as compared to the general population. Further details on sample characteristics are displayed in Table 2.

There were some differences in sociodemographic data between families who participated in FU18 and those who did not (dropouts): the dropouts were more likely to be single parents (*p* = 0.003), the parents were more likely to have a lower high school diploma (*p* < 0.001), the household income was slightly lower (*p* = 0.014), and the mothers were younger (*p* = 0.029). These differences limit the representativeness compared to the initial sample.

### 3.2. Which Factors Are Associated with Young Adults School and Professional Performance at Age 22? Results from Correlation and Regression Models

#### 3.2.1. Bivariate Correlative Analysis

All correlations were in the expected directions (see Table 3). The school-leaving degree of the parents showed a comparatively high correlation with the school degree, overall grade, and professional degree of the young adults. Similarly, household income was significantly associated with the young adults’ school and professional achievements.

The school and professional degree of the young adults at age 22 and the overall school-leaving grade were also significantly associated with the child’s early cognitive capacities and skills (IQ) as well as with the child’s mental health problems at preschool age. Children’s early self-control was also significantly associated with their later school-leaving and professional degree. In contrast, there were no significant associations with maternal dysfunctional parenting or maternal mental health problems in early childhood. Significant correlations, however, were found for adverse childhood events (ACEs), on the one hand, and bullying, on the other hand: young adults with fewer ACEs and no experiences of bullying in childhood or youth reported higher school and professional degrees.

The results of the bivariate analysis can be summarized as follows: a low socioeconomic family background, low child cognitive capacities and skills (IQ), early child mental health problems, adverse childhood experiences, and bullying experiences correspond with lower school and professional success until the age of 22 years.

#### 3.2.2. Regression Analysis

Concerning school and professional degrees, the lowest qualification status was compared with the highest qualification (e.g., no school-leaving certificate or lower secondary school-leaving certificate compared with 12/13 classes, i.e., high school and A-Levels; Table 4), and the intermediate qualification was compared with the highest qualification (e.g., intermediate school-leaving certificate of 10 classes compared with 12/13 classes, i.e., high school and A-Levels; Table 4).

The results of the multinomial logistic hierarchical regression analysis (school-leaving certificate and professional qualification status) and the multiple linear hierarchical regression analysis (overall school-leaving grade) can be summarized as follows (see Table 4, Table 5 and Table 6): as expected, the predictive power of the models improves with the number of variables included. The improvements across the five models are particularly evident for school-leaving degree as well as professional degree and less so for the overall school-leaving grade. Improvements in the model fit appear with the inclusion of early childhood characteristics (Model 1 to Model 2) as well as ACEs and bullying (Model 4 to Model 5). The school-leaving degree was slightly better predicted than the professional degree (QL test, Goodness of Fit, Nagelkerke’s R2, and correct prediction).

The parents’ school-leaving degree proved to be an important predictor, particularly for the prediction of children’s school-leaving degree (Table 4 and Table 5). In contrast to the bivariate comparison, household income had no significant influence. Early learned skills of children rather than fluid intelligence capacities significantly predicted school grades. Children’s sex, early self-control, and early mental health problems as well as early dysfunctional parenting and early mental health problems of the mothers had no significant impact on the young adults’ school or professional success.

In the multinomial comparison between the lowest and the highest school degree (Table 4), adverse childhood experiences (ACEs) and bullying were both identified as significant predictors. At the same time, the parents’ school-leaving degree was no longer significant. This means that ACEs and bullying experiences were more predictive for the young adults’ highest school degree than the parents’ school-leaving degree. This effect was no longer found when comparing the middle and highest school-leaving degree (Table 4, lower part).

In the multinomial comparison between the lowest and the highest professional degree Table 5), ACEs, but not bullying, proved to be significant predictors. At the same time, the influence of the parents’ school-leaving degree remained significant. This means that both ACEs and the parents’ school-leaving degree significantly predicted the young adult’s professional degree. This effect was no longer found in the comparison between the middle and highest professional degree (Table 5, lower part).

In the prediction for overall school-leaving grade (Table 6), besides parents’ school degree, only children’s early learned skills (IQ) appeared to be of additional importance. All other predictors did not make a significant contribution to the prediction model. In addition, young women achieved slightly better grades than young men (small effect-size in models 1 to 4); however, the effect of the young adult’s sex was not significant.

Parental participation in the Triple P parenting intervention at child’s preschool age had no significant effect on the young adults’ professional degree and overall school-leaving grade. Young adults of parents who participated in the Triple P at the beginning of the longitudinal study, however, were a bit more likely to have a high school degree (12/13 classes completed) than a 10 classes school degree (ß = 0.037 **, Table 4).

## 4. Discussion

### 4.1. Predictors for School and Professional Performance in Young Adulthood: Findings from the Longitudinal Study

This 18-year longitudinal study aimed to predict school and professional achievement in young adults by using various context, parental, and child characteristics that were collected in childhood. The results from this investigation on children from age 4 to 22 years old and their families support some findings from earlier research but also bring about some unexpected new findings regarding the prediction of children’s school and professional success.

It was initially assumed that socioeconomic and family characteristics would have a significant impact on children’s school development. The first robust finding of the current investigation is that parents’ educational level significantly predicted their children’s school and professional success 18 years later. This aligns with U.S. findings showing that parents’ educational level when the child was 8 years old significantly predicted the child’s educational and occupational success 40 years later [65]. It is widely known that more educated people are generally healthier than lower educated people (e.g., Wang et al. [66]). More educated parents may thus have better resources for educating their children about coping with life problems, knowledge, and health behavior (e.g., nutrition and mobility) and to support them to learn skills and academic contents at school, which form the basis for starting a successful life. In addition, these parents usually place more value on academic education and, therefore, try to create a modern and adaptable academic environment for their children [67].

Besides the parents’ educational level, other family variables did not have great impact on children’s school and professional achievements until the age of 22 years, i.e., maternal parenting behavior and maternal mental health problems. This may be partly astonishing, since it is well known that mental disorders have a relevant genetic component, and that parental mental health problems significantly predict child mental health (e.g., [68,69]). Mental health problems and school achievements, however, are different things, and apparently children’s school achievement can develop positively even if a parent (here: the mother) has some own mental health problems. Socioeconomic background (assessed by household income) turned out to be only partially predictive. Considering the well-known narrow association between a family’s socioeconomic background and their children’s academic achievements, our finding is partly unexpected. The results illustrate the importance of examining family factors (e.g., parents’ educational level, parenting behavior, and parental mental health) and contextual factors (e.g., household income, ACEs, and bullying experiences) in a very differentiated way. Thus, there is hope that not only context and economic aspects determine school and professional success, but that there are degrees of freedom for positive child developments. A starting point, e.g., could be the child’s learning history outside the core family. Although the core family is of great importance in young childhood, children’s later school performance can also benefit from skills learning in other contexts, such as kindergartens or preschools [70], school environments in urban location with health policies [71], practice-oriented school activities [72], and peer-support [73].

Children’s learned skills—thus behavioral and therefore modifiable aspects of intelligence—turned out to be important cognitive capacities in the children themselves which may be helpful on the way to school and professional achievements. In contrast, children’s early mental health problems (symptoms severity) did not significantly impact school and professional long-term outcome. This finding demonstrates the importance of distinguishing between behavioral skills and learned capacities (what a person can do and activities), on the one hand, and mental health problems (i.e., symptoms and behavioral problems due to mental disorders), on the other hand. (Recurrent) Mental health *symptoms* cannot be prevented by means of environment and parenting [68,69], because they are chronic due to their (partly biologically determined) nature (e.g., [67,68,74,75,76]). *Behavioral and participation problems* (which are often byproducts of mental health symptomatology), however, can be reduced by training and skills learning. For example, a person with a chronic affect dysregulation symptomatology can learn to notice when he or she becomes affectively tense and at risk to react impulsively. He or she can learn to leave the situation in good time before the emotional outburst or learn to cool down by using self-regulation skills. However, this will not prevent him or her from recurring affective tension as such. Our findings indicate that learned skills, on the one hand, and mental health symptomatology, on the other hand, differentially impact child development even in a longitudinal study over 18 years. Mental health symptomatology is often chronic, and skills can be learned and enable people to cope with recurring symptoms.

The multinomial comparisons furthermore showed that adverse childhood experiences (ACEs) in particular, but also bullying experiences in adolescence, significantly predicted school and professional success. These findings are consistent with other results that found significant associations for ACEs and bullying experiences with mental health problems [77,78,79]. However, there are few studies that have investigated the association between ACEs or bullying experiences and school and professional success [79]. In the current investigation, ACEs and bullying experiences were even more important for the prediction of the school-leaving degree than the parents’ school education and socioeconomic background. Individual stressful experiences in childhood and adolescence may thus be important for school achievements. Because there is a wide variety of possible stressful events in childhood and youth, in and out of the core family or at school, it can be assumed that there is hardly one common way for how stressful events may impact child’s school career. Peer conflicts or family trouble at a young age, in some cases, can lead to problematic family communication, the induction of irritation in the child, and resulting problems with learning and school participation. Preventive activities aiming to support coping with unavoidable stress and conflict management may focus, e.g., on functional parenting behavior and resilience training for children: through targeted support, resilience can be strengthened as early as kindergarten age. This can be realized, e.g., by child-centered support programs [80] or parent training with low-threshold access for families with increased risk exposure. Cognitive-behavioral parent training has already been proven to be effective in this context [36].

Longitudinal studies offer a great opportunity to identify long-term effects. However, the results should be interpreted with caution. Even in longitudinal studies, significant influences can actually be due to other variables, especially if there is a longer period of time between surveys. Another problem lies in the generalizability of the results to the present situation. The first assessment (Pre) was conducted in 2001−2003. More than 20 years have passed since then, raising the question to what extent the living conditions of families with young children and the distribution of children in kindergartens have changed. For example, demographic change has led to an increase in the proportion of children with a migration background, technological change has led to new learning methods, e.g., at school, and new academic programs and professions as well as improved childcare options have emerged over the last 20 years. The independent variables we analyzed, however, are fairly universal, and the assessment methods used are likely to remain reliable and valid over time. It would be highly speculative to assume different processes and mechanisms of influence today; nevertheless, social changes and new challenges call for caution and far-reaching conclusions.

### 4.2. Implications for Research

Our aim was to analyze which variables best predict school and professional success, i.e., to find the ‘best’ predictors. Future studies should conduct mediator analyses to examine how parental background affects child factors (e.g., intelligence and mental health) and how this in turn affects school and professional success.

As the study sample is not representative, particularly with regard to socioeconomic status, and the variance is therefore lower than in a representative sample, it can be assumed that the results would certainly be more significant in a representative sample. This would, of course, have to be verified in samples with a larger number of children with a low socioeconomic background.

In addition to maternal variables, future studies should also consider variables from fathers or other caregivers to provide a complete picture of family influences on child school and professional success.

### 4.3. Strengths and Limitations

We analyzed data of a longitudinal study that repeatedly and prospectively assessed family, parental, and individual characteristics of children over 18 years from early childhood to young adulthood. The high retention rate of 80% even after 18 years is a major strength of the present investigation. One reason for the high response rate is the maintenance of intensive contact with the participating families over the whole study period, i.e., reporting results to the families and in the local media, and regular contact with the families, e.g., in terms of Christmas greetings and informational letters. The families furthermore obtained some financial compensation for their effort to participate in the interviews. A lot of families were interested in the longitudinal research themselves, many of them were higher educated and thus may have a sense for the importance of continuing their participation in a longitudinal study. Until FU10, the interviews were conducted in person which may also increase commitment for continued participation. Finally, the families were recruited in the city of research, which may foster the perception of being part of a common project. School success was not only operationalized by the young adults’ school-leaving degree but also by the overall school-leaving grade, and we also considered the current professional degree at age 22 years as another outcome variable for educational success.

Limitations of this study are a rather small sample size of families with a low socioeconomic background (low household income and low highest school-leaving qualification of parents) and a small sample size of families with a migration background; therefore, the representativeness of the current sample is somewhat limited. Furthermore, almost 80% of the young adults had reached a high school degree (12/13 school years completed), and almost 60% had already completed a college degree or were in college. This high level of education may somewhat limit the significance of the results, because obviously less well-educated young adults are underrepresented in the sample, too.

Another limitation is that about half of the families participated in a parent training after the baseline assessment. However, since 18 years have passed, there are multiple other developmental impact factors, which were broadly included in the prediction models; moreover, participation in the parent training was also included in the analysis to control for potential intervention effects.

## 5. Conclusions

Parents’ educational level explains a large part of variance in children’s school and professional success. There are, however, some other aspects of interest: skills and competency aspects may be more predictive for young adults’ school and professional success than early life economic and health conditions of parents and children. This is good news as it supports the idea that health deficits can be partly compensated for by learning and skills training. For children’s development and educational achievements, the findings imply that education, preventive interventions, and treatment should also focus on strengthening behavioral skills rather than just focusing on reducing or even fully erasing mental health symptoms.

Aside from the implications already mentioned, the present results confirm that children from families with low-educated parents in particular deserve special support. It might furthermore be useful to conduct performance tests with all children at an early age to assess their early learned skills. This would allow children with special needs to be identified at an early stage and supported with indicated prevention measures.

As adverse childhood experiences and bullying experiences in childhood and youth may also have a negative impact, but are not always avoidable, preventive interventions should also address coping and resilience and thus support child development with regard to positive school and career achievements.

## Figures and Tables

**Table 1:** Instruments used in the first assessment (Pre), the 10-year follow-up (FU10), and in the 18-year follow-up (FU18).

Predictors and Characteristics	Type of Assessment and Psychometrics	Instruments Name and Content
Socio-demographics (Pre, FU10, FU18)	The sociodemographic interview checklist contains no psychometric contents	Categorical interview checklist exploring: age (childhood and young adulthood), biological sex (child), parenting status (2-parent family vs. single parent), highest school education (mother and father), monthly household income, and migration background
Intelligence—child (Pre)	Intelligence test conducted with child Cronbach’s α for abilities 0.94; Cronbach’s α for skills 0.98	Kaufman Assessment Battery for Children (K-ABC [42]), scales: intellectual abilities and intellectual skills
Self-control problems—child (Pre)	Psychometric questionnaire filled out by mother, each item scaled: 0–2 Retest reliability: 0.80 Split-half reliability: 0.90 Cronbach’s α: 0.93	Child Self-Control Scale [57], 26 items from the domains of lack of control (e.g., “child gets angry quickly”), aggressiveness (e.g., “child gets into scuffles, fights easily”), and hyperactivity (e.g., “child cannot sit still, is restless or overactive”)
Mental health problems—child (Pre)	Psychometric questionnaire filled out by mother, each item scaled: 0–2 Cronbach’s α: 0.94 (mother)	Child Behavior Check List (CBCL 1.5-5 [58]), mother’s report; higher scores = more child behavior problems
Dysfunctional parenting behavior—mother (Pre)	Psychometric questionnaire filled out by mother, each item scaled: 1–7 Retest reliability: 0.84 (mother) Cronbach’s α: 0.80 (mother)	German version of the Parenting Scale (EFB [59]), mother’s report, higher scores = more dysfunctional parenting behavior
Mental health problems—mother (Pre)	Psychometric questionnaire filled out by mother, each item scaled: 1–4 Cronbach’s α: 0.96 (mother)	Depression–Anxiety–Stress Scale (DASS) [60], higher values = more symptom severity
Bullying—adolescent (FU10)	Psychometric questionnaire filled out by child, each item scaled: 1–5 Cronbach’s α: 0.68–0.83	Bully–Victim Questionnaire (BVQ [61]); bullying yes or no
ACE—child (FU18)	Psychometric questionnaire filled out by child, each item scaled: 0–1 Cronbach’s α: 0.76	German version of the Adverse Childhood Experiences Questionnaire (ACE-D [62]), sum score from 10 items (e.g., “Before your 18th birthday: Did a parent or another adult in your household often or very often push you, grab you, hit you or throw something at you?”); higher score = more ACE
School-related data—young adult (FU18)	The sociodemographic interview checklist contains no psychometric contents	Type of school (FU10), school-leaving grades (overall grade, 1.0 = top grade; FU18), school-leaving certificate (FU18), professional certificate (FU18)—explored in interview

**Table 2:** Sample characteristics from Pre to the 18-year follow-up (FU18). N = 225.

Characteristics	M	SD
Age child in years (Pre)	4.00	1.0
Age adolescent in years (FU10)	14.4	1.1
Age young adult in years (FU18)	22.6	1.1
Overall school grade (FU18)Lower grades indicate better school achievements	2.54	0.65
School-leaving degree parents ^1^ (Pre)	2.51	0.63
Household income ^2^ (Pre)	8.74	2.81
IQ fluid capacities (Pre)	103.47	12.11
IQ learned skills (Pre)	102.56	13.76
Self-control problems ^3^ (Pre)	13.89	9.74
Early mental health problems (child) ^4^ (Pre)	48.63	10.24
Dysfunctional parenting (Pre)	3.22	0.56
Parental mental health problems (mother) (Pre)	22.80	16.02
ACE adverse childhood experiences (FU18)	1.03	1.58
	N	%
Sex of the child (Pre)		
- girl	111	49.3
- boy	114	50.7
Parent status (Pre)		
- two-parent family	184	81.8
- mother only	40	17.8
- father only	1	0.4
Education level mother (Pre)		
- without school-leaving certificate. 9 classes	16	7.1
- 10 classes	78	34.8
- A-Levels/High school	130	58.0
Education level father (Pre)		
- without school-leaving certificate. 9 classes	21	11.4
- 10 classes	47	25.5
- A-Levels/High school	116	63.0
Monthly household income (EUR/USD) (Pre)		
- <1.000	1	0.5
- 1.000 to <1.500	5	2.3
- 1.500 to <2.000	4	1.8
- 2.000 to <2.500	19	8.7
- 2.500 to <3.000	6	2.7
- 3.000 to <3.500	14	6.4
- 3.500 to <4.000	15	6.8
- 4.000 to <4.500	20	9.1
- 4.500 to <5.000	18	8.2
- 5.000 to <6.000	44	20.1
- 6.000 to <8.000	44	20.1
- 8.000 to <10.000	26	11.9
- ≥10.000	3	1.4
Migration background (Pre)		
- with	24	10.7
- without	201	89.3
Bullying (FU10)		
- bullying	47	22.2
- no bullying	165	77.8
School-leaving certificate (young adult. FU18)		
- no school-leaving degree	3	1.3
- special school-leaving degree	1	0.4
- 9 classes school-leaving degree	11	4.9
- 10 classes school-leaving degree	30	13.4
- 12/13 classes A-Levels/high school	179	79.9
Professional certificate (young adult. FU18)		
- no professional certificate, not in any professional education	31	14.0
- currently in professional education	19	8.6
- professional certificate	39	17.6
- college/university studies	101	45.5
- college/university certificate	32	14.4

*Note.* ^1^ 3-stepped scale: 1 = no school-leaving degree, 9 classes, 2 = 10 classes school-leaving degree, and 3 = 12/13 classes A-Levels; mean of mother (and if available father) school-leaving degree; ^2^ 13-stepped scale: 1 < 1.000 DM to 13 > 10.000 DM); ^3^ high values = low self-control; ^4^ t-values.

**Table 3:** Means, standard deviations, and correlations of study variables (Pre, FU10, and FU18).

**Characteristics**	**N**	**N**	**%**	**School-Leaving Degree ^5^**	**Professional Degree ^5^**	**Overall School Grade ^6^**
School-leaving degree (FU18)	224			-	0.675 ***	−0.169 **
- no school-leaving degree. 9 classes		15	6.7			
- 10 classes school-leaving degree		30	14.4			
- 12/13 classes A-Levels/high school		179	79.9			
Professional degree (FU18)	222			0.675 ***	-	−0.270 ***
- without professional education		31	14.0			
- in professional education		58	26.1			
- in college/university studies		133	59.9			
Biological sex	225			0.098	0.088	0.111
- female		111	49.3			
- male		114	50.7			
Parenting status (Pre)	225			0.128	0.103	0.095
- 2-parent family		184	81.8			
- single parent		41	18.2			
Parental training Triple P (Pre)	225			0.085	0.094	−0.072
- no participation		104	46.2			
- participation		121	53.8			
Bullying (FU10)	212			0.365 ***	0.211 **	−0.192 **
- bullying		165	77.8			
- no bullying		47	22.2			
**Characteristics**	**N**	**M**	**SD**	**School-leaving degree ^6^**	**Professional degree ^6^**	**Overall school grade ^7^**
Overall school grade (FU18) Lower grades indicate better school achievements	215	2.54	0.65	−0.169 **	−0.270 ***	-
School-leaving degree parents ^1^ (Pre)	224	2.51	0.63	0.363 ***	0.291 ***	−0.288 ***
Household income ^2^ (Pre)	219	8.74	2.81	0.183 ***	0.223 ***	−0.216 **
IQ fluid capacities (Pre)	218	103.47	12.11	0.253 ***	0.211 ***	−0.290 ***
IQ learned skills (Pre)	220	102.56	13.76	0.257 ***	0.237 ***	−0.292 ***
Self-control problems ^3^ (Pre)	223	13.89	9.74	−0.169 **	−0.155 **	0.072
Early mental health problems (child) ^4^ (Pre)	223	48.63	10.24	−0.161 **	−0.150 **	0.138 *
Dysfunctional parenting (Pre)	223	3.22	0.56	−0.106	−0.083	0.105
Parental mental health problems (mother) (Pre)	223	22.80	16.02	−0.058	−0.107 *	0.115
ACE	211	1.03	1.58	−0.164 **	−0.161 **	0.067

*Note.* *** *p* < 0.001, ** *p* < 0.05, * *p* < 0.01; all tests were one sided, test for sex was 2-sided; ^1^ 3-stepped scale: 1 = no school-leaving degree, 9 classes, 2 = 10 classes school-leaving degree, and 3 = 12/13 classes A-Levels; mean of mother (and if available father) school-leaving degree; ^2^ 13-stepped scale: 1 < 1.000 DM to 13 > 10.000 DM); ^3^ high values = low self-control; ^4^ t-values; ^5^ φ coefficient; ^6^ Kendall τ; ^7^ Spearman r_s_.

**Table 4:** Prediction of school-leaving degree (FU18): multinomial logistic regression (inclusion method, hierarchical).

**Dependent Variable: Without School-Leaving Degree or 9 Classes Versus 12/13 Classes**															
**Predictor**	**Model 1**			**Model 2**			**Model 3**			**Model 4**			**Model 5**		
	**B**	***p***	**d**	**B**	***p***	**d**	**B**	***p***	**d**	**B**	***p***	**d**	**B**	***p***	**d**
School-leaving degree parents ^1^ (Pre)	−2.068	<0.001 ***	−1.14 ^###^	−1.416	0.032 *	−0.78 ^###^	−1.585	0.031 *	−0.87 ^###^	−1.625	0.027 *	−0.90 ^###^	−3.001	0.096	−1.65 ^###^
Household income ^2^ (Pre)	−0.257	0.023 *	−0.14	−0.304	0.051	−0.17	−0.364	0.033 *	−0.20 ^#^	−0.323	0.073	−0.18	0.077	0.807	0.04
Sex of the child	0.071	0.910	0.04	−0.303	0.718	−0.17	−0.373	0.675	−0.21 ^#^	−0.417	0.643	−0.23 ^#^	0.498	0.781	0.27 ^#^
IQ Fluid capacities (Pre)				−0.096	0.042 *	−0.05	−0.093	0.057	−0.05	−0.089	0.069	−0.05	−0.149	0.140	−0.08
IQ Learned skills (Pre)				−0.026	0.490	−0.13	−0.041	0.290	−0.02	−0.043	0.275	−0.02	−0.098	0.297	−0.05
Self-control problems ^3^ (Pre)				0.075	0.205	−0.04	0.092	0.152	0.05	0.090	0.164	0.05	0.138	0.253	0.08
Mental health problems (child) ^4^ (Pre)				−0.013	0.832	−0.01	−0.018	0.792	−0.01	−0.010	0.884	−0.01	0.008	0.934	0.00
Dysfunctional parenting (Pre)							0.395	0.654	0.22 ^#^	0.475	0.610	0.26	0.177	0.898	0.10
Parental mental health problems (mother) (Pre)							−0.036	0.085	−0.02	−0.037	0.088	−0.02	−0.062	0.292	−0.03
Parental training (Triple P) participation (Pre)										−0.740	0.428	−0.41 ^#^	1.723	0.356	−0.95 ^###^
ACE adverse childhood experiences (FU18)													1.006	0.016 *	0.55 ^##^
Bullying (FU10)													−2.230	0.037 *	−1.23 ^###^
**Dependent variable: School-leaving degree after 10 classes versus after 12/13 classes**															
**Predictor**	**Model 1**			**Model 2**			**Model 3**			**Model 4**			**Model 5**		
	**B**	***p***	**d**	**B**	***p***	**d**	**B**	***p***	**d**	**B**	***p***	**d**	**B**	***p***	**d**
School-leaving degree parents ^1^ (Pre)	−1.408	<0.001 ***	−0.78 ^##^	−1.515	<0.001 ***	−0.83 ^###^	−1.546	<0.001 ***	−0.85 ^###^	−1.610	<0.001 ***	−0.89 ^###^	−1.837	<0.001 ***	−1.01 ^###^
Household income ^2^ (Pre)	0.019	0.820	0.01	0.066	0.515	0.04	0.050	0.628	0.03	0.079	0.457	0.04	0.120	0.350	0.07
Sex of the child	0.771	0.080	0.43 ^#^	0.672	0.167	0.37 ^#^	0.682	0.165	0.38 ^#^	0.636	0.200	0.35 ^#^	0.822	0.187	0.45 ^#^
IQ Fluid capacities (Pre)				0.005	0.846	0.00	0.006	0.802	0.00	0.008	0.771	0.00	−0.019	0.537	−0.01
IQ Learned skills (Pre)				−0.032	0.165	−0.02	−0.034	0.135	−0.02	−0.035	0.133	−0.02	−0.013	0.617	−0.01
Self-control problems ^3^ (Pre)				0.000	0.997	0.000	0.006	0.878	0.00	0.010	0.806	0.01	0.036	0.441	0.02
Mental health problems (child) ^4^ (Pre)				0.011	0.782	0.01	0.015	0.711	0.01	0.022	0.581	0.01	0.030	0.523	0.02
Dysfunctional parenting (Pre)							−0.199	0.685	−0.11	−0.278	0.581	−0.15	−0.353	0.541	−0.19
Parental mental health problems (mother) (Pre)							−0.008	0.579	−0.00	−0.009	0.575	−0.01	−0.014	0.389	−0.01
Parental training (Triple P) participation (Pre)										−0.791	0.106	−0.44 ^#^	−10.260	0.037 *	−0.69 ^##^
ACE adverse childhood experiences (FU18)													0.300	0.167	0.17
Bullying (FU10)													−0.133	0.545	−0.07
LQ-Test	χ^2^ = 52.1, *p* < 0.001			χ^2^ = 73.5, *p* < 0.001			χ^2^ = 77.2, *p* < 0.001			χ^2^ = 80.1, *p* < 0.001			χ^2^ = 76.6, *p* < 0.001		
Goodness of Fit	χ^2^ = 77.4, *p* = 0.918			χ^2^ = 176.4, *p* = 1.000			χ^2^ = 173.2, *p* = 1.000			χ^2^ = 170.3, *p* = 1.000			χ^2^ = 117.9, *p* = 1.000		
Nagelkerkes *R*^2^	*R*^2^ = 0.297			*R*^2^ = 0.426			*R*^2^ = 0.443			*R*^2^ = 0.457			*R*^2^ = 0.552		
Correct prediction	82.5%			86.5%			87.0%			86.5%			87.5%		

*Note.* *** *p* < 0.001, * *p* < 0.01; ^#^ > 0.20, <−0.20 small effect, ^##^ > 0.50, <−0.50 medium effect, ^###^ > 0.80, <−0.80 large effect; ^1^ 3-stepped scale: 1 = no school-leaving degree, 9 classes, 2 = 10 classes school-leaving degree, and 3 = 12/13 classes A-Levels; mean of mother (and if available father) school-leaving degree; ^2^ 13-stepped scale: 1 < 1.000 DM to 13 > 10.000 DM; ^3^ high values = low self-control; ^4^ t-values.

**Table 5:** Prediction of professional degree (FU18): multinomial logistic regression (inclusion method, hierarchical).

**Dependent Variable: Without Professional Education Versus College/University**															
**Predictor**	**Model 1**			**Model 2**			**Model 3**			**Model 4**			**Model 5**		
	***B***	***p***	***d***	***B***	***p***	***d***	***B***	***p***	***d***	***B***	***p***	***d***	***B***	***p***	***d***
School-leaving degree parents ^1^ (Pre)	−1.041	0.002 **	−0.57 ^##^	−0.965	0.013 *	−0.53 ^###^	−1.081	0.007 **	−0.60 ^##^	−1.164	0.004 **	−0.64 ^##^	−1.356	0.009 **	−0.758 ^##^
Household income ^2^ (Pre)	−0.169	0.030 *	−0.09	−0.171	0.050	−0.09	−0.199	0.027 *	0.11	−0.169	0.069	−0.09	0.034	0.784	0.02
Sex of the child	0.644	0.138	0.36 ^#^	0.667	0.165	0.37 ^#^	0.672	0.168	0.37 ^#^	0.639	0.201	0.35 ^#^	20.043	0.011 *	10.13 ^###^
IQ Fluid capacities (Pre)				−0.012	0.620	−0.01	−0.008	0.768	−0.00	−0.005	0.849	−0.00	0.016	0.603	0.01
IQ Learned skills (Pre)				−0.023	0.308	−0.01	−0.029	0.197	−0.02	−0.029	0.199	−0.02	−0.010	0.721	−0.01
Self-control problems ^3^ (Pre)				0.028	0.458	0.02	0.043	0.274	0.02	0.050	0.217	0.03	0.012	0.805	0.01
Mental health problems (child) ^4^ (Pre)				0.010	0.789	0.01	0.019	0.636	0.01	0.023	0.562	0.01	0.074	0.157	0.04
Dysfunctional parenting (Pre)							−0.409	0.387	−0.23	−0.495	0.309	−0.27 ^#^	−0.588	0.319	−0.32 ^#^
Parental mental health problems (mother) (Pre)							−0.024	0.181	−0.01	−0.024	0.184	−0.01	−0.025	0.288	−0.01
Parental training (Triple P) participation (Pre)										−0.913	0.067	−0.50 ^##^	−0.813	0.174	−0.45 ^#^
ACE adverse childhood experiences (FU18)													0.804	<0.001 ***	0.45 ^#^
Bullying (FU10)													−0.291	0.157	−0.16
**Dependent variable: professional education versus college/university**															
**Predictor**	**Model 1**			**Model 2**			**Model 3**			**Model 4**			**Model 5**		
	***B***	***p***	***d***	***B***	***p***	***d***	***B***	***p***	***d***	***B***	***p***	***d***	***B***	***p***	***d***
School-leaving degree parents ^1^ (Pre)	−0.939	0.001 ***	−0.52 ^##^	−0.702	0.027 *	−0.39 ^#^	−0.708	0.030 *	−0.39 ^#^	−0.719	0.028 *	−0.40 ^#^	−0.569	0.119	−0.32 ^#^
Household income ^2^ (Pre)	−0.109	0.090	−0.06	−0.091	0.199	−0.05	−0.096	0.183	−0.05	−0.092	0.211	−0.05	−0.141	0.096	−0.08
Sex	0.207	0.540	0.11	0.090	0.806	0.05	0.076	0.837	0.04	0.044	0.905	0.02	−0.062	0.877	−0.03
IQ Fluid capacities (Pre)				−0.011	0.573	−0.01	−0.011	0.569	−0.01	−0.011	0.581	−0.01	−0.013	0.525	−0.01
IQ Learned skills (Pre)				−0.037	0.032 *	−0.02	−0.037	0.035 *	−0.02	−0.038	0.033 *	−0.02	−0.046	0.027	−0.03
Self-control problems ^3^ (Pre)				0.046	0.137	0.03	0.046	0.143	0.03	0.048	0.131	0.03	0.062	0.069	0.03
Mental health problems (child) ^4^ (Pre)				−0.028	0.347	−0.02	−0.026	0.393	−0.01	−0.025	0.413	−0.01	−0.036	0.299	−0.02
Dysfunctional parenting (Pre)							−0.113	0.752	−0.06	−0.132	0.712	−0.07	−0.058	0.881	−0.03
Parental mental health problems (mother) (Pre)							−0.001	0.937	−0.00	−0.001	0.920	−0.00	−0.009	0.520	−0.01
Parental training (Triple P) participation (Pre)										−0.178	0.631	−0.10	−0.067	0.872	−0.04
ACE adverse childhood experiences (FU18)													−0.222	0.259	−0.12
Bullying (FU10)													−0.016	0.926	−0.01
LQ-Test	χ^2^ = 31.4, *p* < 0.001			χ^2^ = 47.6, *p* < 0.001			χ^2^ = 51.9, *p* < 0.001			χ^2^ = 54.9, *p* < 0.001			χ^2^ = 74.4, *p* < 0.001		
Goodness of Fit	χ^2^ = 128.6, *p* = 0.015			χ^2^ = 333.0, *p* < = 0.991			χ^2^ = 329.1, *p* = 0.991			χ^2^ = 325.6, *p* = 0.992			χ^2^ = 260.0, *p* = 1.000		
Nagelkerkes *R*^2^	*R*^2^ = 0.161			*R*^2^ = 0.245			*R*^2^ = 0.262			*R*^2^ = 0.278			*R*^2^ = 0.397		
Correct prediction	62.8%			65.0%			66.5%			65.0%			68.5%		

*Note.* *** *p* < 0.001, ** *p* < 0.05, * *p* < 0.01; ^#^ > 0.10 small effect, ^##^ > 0.30 medium effect, ^###^ > 0.50 large effect; ^1^ 3-stepped scale: 1 = no school-leaving degree, 9 classes, 2 = 10 classes school-leaving degree, and 3 = 12/13 classes A-Levels; mean of mother (and if available father) school-leaving degree; ^2^ 13-stepped scale: 1 < 1.000 DM to 13 > 10.000 DM); ^3^ high values = low self-control; ^4^ t-values.

**Table 6:** Prediction of school grade (FU18): multiple linear regression (inclusion method. hierarchical).

	Model 1		Model 2		Model 3		Model 4		Model 5	
Predictors	*β*	*p*	*β*	*p*	*β*	*p*	*β*	*p*	*β*	*p*
School-leaving degree parents ^1^ (Pre)	−0.329 ^##^	<0.001 ***	−0.251 ^#^	0.001 ***	−0.252 ^#^	0.001 ***	−0.254 ^#^	0.001 ***	−0.267 ^#^	<0.001 ***
Household income ^2^ (Pre)	−0.118 ^#^	0.107	−0.062	0.411	−0.060	0.433	−0.054	0.495	−0.077	0.335
Sex of the child	0.109 ^#^	0.120	0.113 ^#^	0.108	0.113 ^#^	0.111	0.110 ^#^	0.127	0.077	0.286
IQ Fluid capacities (Pre)			−0.009	0.915	−0.011 ^#^	0.903	−0.011 ^#^	0.904	−0.011	0.895
IQ Learned skills (Pre)			−0.236 ^#^	0.008 **	−0.235 ^#^	0.009 **	−0.236 ^#^	0.009 **	−0.240 ^#^	0.008 **
Self-control problems ^3^ (Pre)			−0.075	0.507	−0.074	0.517	−0.071	0.537	−0.065	0.568
Mental health problems (child) ^4^ (Pre)			0.088	0.447	0.087	0.466	0.092	0.445	0.071	0.551
Dysfunctional parenting (Pre)					−0.023	0.767	−0.025	0.743	−0.021	0.782
Parental mental health problems (mother) (Pre)					0.019	0.814	0.017	0.831	−0.005	0.953
Parenting training (Triple P) participation (Pre)							−0.029	0.687	−0.025	0.736
ACE adverse childhood experiences (FU18)									−0.117 ^#^	0.121
Bullying (FU10)									−0.118 ^#^	0.102
	*R* ^2^	0.151	*R* ^2^	0.203	*R* ^2^	0.203	*R* ^2^	0.204	*R* ^2^	0.228
	*corrected R* ^2^	0.137	*corrected R* ^2^	0.170	*corrected R* ^2^	0.161	*corrected R* ^2^	0.157	*corrected R* ^2^	0.173
	*p*	<0.001 ***	*p*	<0.001 ***	*p*	<0.001 ***	*p*	<0.001 ***	*p*	<0.001 ***
	*Change in R* ^2^	0.151	*Change in R* ^2^	0.052	*Change in R* ^2^	0.001	*Change in R* ^2^	0.001	*Change in R* ^2^	0.024
	*Change in p*	<0.001 ***	*Change in p*	0.028 *	*Change in p*	0.939	*Change in p*	0.687	*Change in p*	0.076
	Durbin-Watson- Statistik	2.015	Durbin-Watson- Statistik	2.105	Durbin-Watson- Statistik	2.105	Durbin-Watson- Statistik	2.105	Durbin-Watson- Statistik	2.105
	VIF	1.01–1.101	VIF	1.06–2.88	VIF	1.07–3.03	VIF	1.09–3.06	VIF	1.12–3.09

*Note.* *** *p* < 0.001, ** *p* < 0.05, * *p* < 0.01; ^#^ > 0.10 small effect, ^##^ > 0.30 medium effect; VIF = variance-inflation factor; ^1^ 3-stepped scale: 1 = no school-leaving degree, 9 classes, 2 = 10 classes school-leaving degree, and 3 = 12/13 classes A-Levels; mean of mother (and if available father) school-leaving degree; ^2^ 13-stepped scale: 1 < 1.000 DM to 13 > 10.000 DM); ^3^ high values = low self-control; ^4^ t-values.

## Data Availability

The datasets generated and/or analyzed during the current study are not publicly available as they contain sensitive material. Furthermore, it is a longitudinal study with several assessment points, so that the data could possibly be used to draw conclusions about individuals. The questionnaires used can be found in the corresponding references.
